# Incomplete Radiology Requests Are the Norm: A Quality Audit at a Moroccan Tertiary Hospital

**DOI:** 10.7759/cureus.98353

**Published:** 2025-12-02

**Authors:** El Hajjami Ayoub, Bouktib Youssef, Badr Boutakioute, Meriem Ouali Idrissi, Najat Cherif Idrissi El Ganouni

**Affiliations:** 1 Radiology, CHU (Centre Hospitalier Universitaire) Mohammed VI, Arrazi Hospital, Cadi Ayyad University, Marrakech, MAR

**Keywords:** administrative completeness, clinical information, ct scan, diagnostic accuracy, haute autorité de santé, imaging requisition, medical forms, patient safety, quality audit, radiology requests

## Abstract

Radiology requests initiate the diagnostic process and are a key touchpoint between clinicians and radiologists. Their clarity and completeness directly affect diagnostic precision, patient safety, and resource use. This study evaluated 400 radiology requisition forms, 165 ultrasound, 165 CT, and 70 MRI, at a major tertiary center in Marrakech, Morocco, using eight criteria from the French Haute Autorité de Santé (HAS). While 82% (n=328) met the minimum quality threshold (≥6 of 8 criteria), gaps were evident: only 67% (n=268) included the examination’s clinical purpose, and prior imaging was referenced in just 35% (n=140). CT forms scored highest in conformity (87%, n=144), followed by MRI (82%, n=57) and ultrasound (77%, n=127). Administrative sections were more consistently completed than clinical fields. These findings reveal a systemic need for structured electronic forms, mandatory clinical fields, and physician training to strengthen diagnostic quality and reduce unnecessary imaging.

## Introduction

Radiology examination requests constitute the foundation of diagnostic imaging workflows and serve as the primary channel of communication between referring physicians and radiologists. Their clarity, completeness, and clinical relevance directly influence diagnostic accuracy, patient safety, and the efficient use of imaging resources.

The rapid expansion of imaging technologies has led to a substantial rise in the number of radiological examinations performed. Although this increase reflects enhanced diagnostic capability, it simultaneously raises concerns about the appropriateness, quality, and standardization of imaging requests [1]. Incomplete, unjustified, or ambiguous requisitions may result in unnecessary radiation exposure, higher healthcare costs, and delays in patient management [2,3].

To mitigate these issues, national and international authorities, such as the French Haute Autorité de Santé (HAS), have established standardized criteria for assessing the completeness and quality of radiology request forms [4]. These criteria encompass both administrative and clinical elements. However, adherence to these standards remains inconsistent, particularly in high-demand hospital environments [5].

This study examines the quality of imaging requests submitted to a tertiary care center in Marrakech, Morocco. The objectives were to evaluate compliance with HAS standards using eight defined criteria (percentage values are consistently reported with corresponding frequencies), to assess the completeness of key clinical indications provided by referring physicians, to identify documentation patterns across imaging modalities (CT, MRI, and ultrasound) and referral sources (emergency, inpatient, and outpatient), and to propose practical recommendations aimed at improving the clarity, relevance, and operational quality of radiology requisitions.

## Materials and methods

This was a prospective, descriptive, and analytical study conducted over a three-month period in the Radiology Department of Arrazi Hospital, which is part of the Mohammed VI University Hospital Center in Marrakech, Morocco. A total of 400 imaging requests were analyzed, including 165 (41.25%) for ultrasound, 165 (41.25%) for CT, and 70 (17.50%) for MRI. The study was approved by the Ethics Committee for Medical Research, Faculty of Medicine and Pharmacy of Marrakech (approval number: CERB/FMPM/2025/041). All data were handled in compliance with ethical research standards, ensuring full respect for patient anonymity and confidentiality. No identifying information was collected or disclosed at any stage of the study.

Study population and sampling

The study sample comprised 400 imaging requisitions proportionally distributed across the three imaging modalities according to their relative frequency within the department’s overall activity. Based on an estimated annual imaging workload of 50,000 examinations across the Arrazi, Mother and Child, and Ibn Tofail hospital sites, the sample size was determined in collaboration with the Department of Epidemiology, assuming a 5% margin of error and a 95% confidence level. The distribution of the sample according to imaging modality is presented in Table 1.

**Table 1:** Distribution of the sample according to imaging modality

Imaging Modality	Proportion (%)	Sample Size
Ultrasound	41.25	165
CT	41.25	165
MRI	17.50	70
Total	100	400

Only imaging requests for ultrasound, CT, and MRI were included, encompassing those originating from inpatient medical and surgical wards, emergency departments, and private-sector referrals. Requests involving other imaging modalities were excluded. A random sampling method was applied, selecting examination forms from three consecutive days at the beginning of each week to minimize prescription bias related to the same practitioners. Each selected request form was photographed and subsequently analyzed.

Data collection

Data collection was performed using a standardized data sheet. When a single patient had multiple imaging examinations of different modalities (for example, ultrasound and CT), each exam was counted separately. Conversely, when a single request involved multiple anatomical regions of the same modality, it was recorded as a single requisition.

Variables and definitions

Each imaging request was evaluated according to the conformity criteria of the HAS, which include eight core variables. Five of these variables correspond to administrative information: date of request, requesting department, physician’s name, patient identification, and patient date of birth, while the remaining three variables represent clinical information, namely the anatomical region to be imaged, the clinical indication or relevant medical history, and the specific purpose or clinical question guiding the examination.

Statistical analysis

Data were entered and analyzed using Microsoft Excel 2021 (Microsoft Corporation, Redmond, Washington, United States), Microsoft Word 2021 (Microsoft Corporation), and IBM SPSS Statistics for Windows, version 26 (Released 2018; IBM Corp., Armonk, New York, United States). Descriptive statistics were used to summarize the distribution of qualitative and quantitative variables, while analytical methods were applied to assess the conformity and relevance of imaging requisitions according to the HAS criteria.

## Results

A total of 400 radiology examination requests were analyzed. Ultrasound and CT each represented 41.25% (n = 165) of the total sample, while MRI accounted for 17.5% (n = 70). This distribution reflected the typical activity profile of the radiology department. Most forms were of acceptable material and legibility quality. A total of 315 forms (78.8%) were printed on good-quality paper, and 332 (83.0%) were clearly legible. Paper quality and readability were highest in CT forms (88%, n = 145) and lowest in ultrasound forms (76%, n = 125), a difference that reached statistical significance (χ² = 6.47, p = 0.041) (Table 2).

**Table 2:** Administrative and clinical completeness of radiology request forms by imaging modality Values are presented as n (%) unless otherwise specified; p-values were calculated using the Chi-square (χ²) test for categorical variables and one-way analysis of variance (ANOVA) for continuous variables (mean conformity score). HAS: Haute Autorité de Santé

Quality Parameter	Ultrasound (n=165), n (%)	CT (n=165), n (%)	MRI (n=70), n (%)	Total (n=400), n (%)	p-Value
Legible form	130 (79)	145 (88)	57 (81)	332 (83)	0.128
Date indicated	130 (79)	140 (85)	58 (83)	328 (82)	0.311
Physician name present	110 (67)	150 (91)	64 (91)	324 (81)	< 0.00
Physician qualification indicated	125 (76)	130 (79)	45 (64)	300 (75)	0.072
Physician stamp/signature	110 (67)	150 (91)	52 (74)	312 (78)	0.002
Department of origin identified	115 (70)	150 (91)	51 (73)	316 (79)	0.004
Patient name present	165 (100)	158 (96)	68 (97)	388 (97)	0.083
Patient age recorded	147 (89)	137 (83)	59 (84)	340 (85)	0.215
Hospital identification number present	115 (70)	107 (65)	32 (45)	256 (64)	0.003
Anatomical region specified	145 (88)	155 (94)	70 (100)	370 (92)	0.026
Clinical indication/history provided	140 (85)	152 (92)	68 (97)	360 (90)	0.009
Prior imaging referenced	48 (29)	65 (39)	27 (39)	140 (35)	0.118
Urgency level indicated	85 (52)	92 (56)	33 (47)	210 (52)	0.347
Purpose/clinical question stated	102 (62)	115 (70)	51 (73)	268 (67)	0.092
Conformity score, mean ± SD (/8)	6.2 ± 1.1	6.9 ± 0.8	6.6 ± 0.9	6.7 ± 1.1	0.030
Overall HAS conformity ≥ 6 /8	127 (77)	144 (87)	57 (82)	328 (82)	0.046

Administrative completeness varied significantly among imaging modalities. The date of request appeared in 82% (n = 328) of all forms, the department of origin in 79% (n = 316), and the physician’s name in 81% (n = 324). The prescriber’s qualification or title was indicated in 75% (n = 300), and a valid stamp or signature was present in 78% (n = 312). CT requests were significantly more complete than MRI or ultrasound requests, with a mean administrative conformity score of 6.9 ± 0.8 compared with 6.6 ± 0.9 and 6.2 ± 1.1, respectively (one-way ANOVA F = 3.54, p = 0.032).

Patient identification data were generally satisfactory. The patient’s name appeared in 97% (n = 388) of forms, age in 85% (n = 340), and hospital identification number in 64% (n = 256). The hospital ID field was least frequently completed in MRI requests (45%, n = 32), reflecting their greater outpatient or private-sector origin (χ² = 11.43, p = 0.003). The mean patient-identification completeness score was highest for ultrasound (2.6 ± 0.6) compared with CT (2.4 ± 0.7) and MRI (2.1 ± 0.8) (ANOVA p = 0.021).

Request origin analysis showed that 43% (n = 172) of requisitions came from emergency departments, 38% (n = 152) from inpatient wards, and 19% (n = 76) from external or private sources. The indication of urgency appeared in 52% (n = 208) of all forms and was strongly associated with emergency requests (χ² = 22.4, p < 0.001) (Table 2).

Clinical content was generally more robust. The anatomical region was specified in 92% (n = 368) of requests, a clinical history or indication in 90% (n = 360), and the imaging modality in 98% (n = 392). Prior imaging studies were mentioned in only 35% (n = 140), while the specific diagnostic question or objective appeared in 67% (n = 268). MRI requests contained the most comprehensive clinical information, with a mean clinical completeness score of 6.3 ± 1.0, followed by CT (5.9 ± 1.2) and ultrasound (5.4 ± 1.3) (ANOVA F = 4.27, p = 0.015).

**Figure 1:**
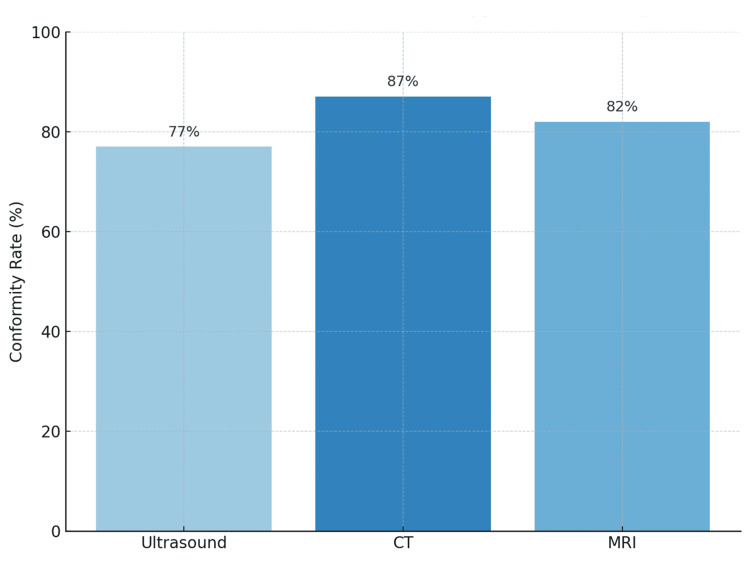
Overall HAS conformity rate by imaging modality CT exhibited the highest conformity (87%, n=144), followed by MRI (82%, n=57) and ultrasound (77%, n=127). HAS: Haute Autorité de Santé

Global conformity with the HAS quality criteria, defined as documentation of at least six of eight required parameters, was achieved in 82% of all requests. CT exhibited the highest overall conformity (87%, n = 144), followed by MRI (82%, n = 57) and ultrasound (77%, n = 127) (χ² = 6.17, p = 0.046). The mean overall conformity score (maximum = 8) was 6.7 ± 1.1 across all forms, differing by modality as follows: CT = 6.9 ± 0.8, MRI = 6.6 ± 0.9, and ultrasound = 6.2 ± 1.1 (ANOVA p = 0.03) (Figure 1, Table 2).

## Discussion

This study reveals significant variability in the quality of radiology examination requests submitted to Arrazi Hospital, despite a generally high overall compliance rate with HAS standards (82%). While administrative fields such as patient name (97%) and physician identification (81%) were consistently completed, critical clinical components, including the diagnostic purpose (absent in 33% of cases) and reference to prior imaging (missing in 65%), were often omitted.

This trend reflects a common issue in medical imaging workflows: administrative information is frequently prioritized over clinical context. While radiologists rely on comprehensive clinical details to select protocols and interpret findings accurately, referring clinicians may focus primarily on fulfilling procedural requirements. The imbalance contributes to suboptimal communication and increases the risk of inappropriate or redundant imaging. Similar trends have been observed in studies by Roussel and Lelièvre [5] and Cohen et al. [6], where administrative fields were completed more consistently than clinical sections. The implication is that while basic logistical information is often present, the nuanced clinical context essential for optimal radiological interpretation and patient care remains frequently deficient [7]. This deficiency often necessitates additional communication between radiologists and referring clinicians, introducing delays and potential inefficiencies in the diagnostic process [8]. Such delays can be particularly detrimental in urgent cases, where timely and accurate diagnosis directly impacts patient outcomes.

Among the three modalities analyzed, CT requests had the highest overall conformity (87%), likely due to better integration with hospital systems and more structured protocols. Ultrasound requests showed the lowest quality scores (77%), which may be attributed to higher patient turnover, more variable requesters (e.g., general practitioners), and limited use of standardized forms. MRI requests, often tied to specialized services, included more comprehensive clinical information. This suggests that modalities with more specialized indications or those integrated into established referral pathways tend to elicit more thorough clinical details, underscoring the influence of system design and clinical context on request completeness [9,10]. Therefore, efforts to standardize and improve the completeness of radiology requests should consider the specific characteristics and typical workflows associated with each imaging modality to ensure optimal diagnostic pathways [11].

Although digital tools such as Radiology Information Systems (RIS) and Electronic Health Records (EHR) are increasingly available, their effectiveness depends on how well they are implemented [7,8]. The presence of electronic forms alone does not ensure data quality. Many forms remain partially structured or rely on free-text inputs, leading to inconsistencies and missing fields [12]. Requests originating from emergency departments, responsible for over 40% of submissions, were frequently incomplete, likely due to time constraints and high workloads. These departments represent a key area for improvement, as imaging decisions in emergency care carry heightened clinical and operational consequences [13]. To improve request quality, healthcare institutions should implement structured digital requisition systems with mandatory fields for clinical information such as exam purpose, prior imaging, and clinical indication [14]. These systems should be linked to patient records to enable autofill of administrative data, reducing manual errors and duplication.

In parallel, regular audits and feedback loops between radiology departments and referring units can reinforce accountability and track compliance over time. Education and sensitization programs for physicians, especially junior doctors and emergency department staff, should focus on the clinical and operational impact of complete imaging requests.

This study has several limitations. It was conducted at a single tertiary institution and may not reflect practices in smaller hospitals or private clinics. Moreover, while it evaluated the formal completeness of requests, it did not assess the clinical appropriateness or diagnostic yield of the imaging studies. Future research should explore the relationship between request quality and diagnostic accuracy, and test the impact of digital interventions such as clinical decision support tools [15,16].

Furthermore, investigating the efficacy of artificial intelligence in analyzing free-text clinical information within requests could mitigate the burden on clinicians while enhancing the precision and completeness of diagnostic imaging referrals. Such technological advancements could aid in identifying critical patient data that might otherwise be overlooked, thereby improving the overall quality and utility of radiological examinations [17]. Further, integrating AI tools that assess the textual quality and clinical relevance of imaging reports could help standardize communication between radiologists and referring physicians [18,19].

## Conclusions

Despite high overall conformity to HAS standards, key clinical information was found to be often missing from radiology requests at Arrazi Hospital, especially the diagnostic purpose and history of prior imaging. These omissions can lead to inappropriate imaging, delayed diagnoses, and inefficient use of resources. To address this, hospitals should adopt structured digital requisition systems that enforce mandatory clinical fields and enable integration with patient records. Regular audits and targeted training for referring physicians are also essential. Improving the quality of radiology requests is not just a documentation issue; it is a necessary step toward safer, faster, and more effective patient care.
